# From polar bears to cell polarity and everything in between: 2018 in review

**DOI:** 10.1242/bio.042119

**Published:** 2019-02-15

**Authors:** Rachel Hackett

**Affiliations:** The Company of Biologists, Bidder Building, Station Road, Cambridge CB24 9LF, UK

It was all change in the BiO Editor team in 2018, with Steven Kelly replacing departing founding Editor-in-Chief Jordan Raff (Kelly, 2018; Raff, 2018). We were delighted to announce the appointment of Tristan Rodríguez (Imperial College London, UK) and Yong Peng (Sichuan University, China) to our international board of research-active Editors. Tristan's research group focuses on understanding the signalling mechanisms that control cell fitness during embryonic development. Yong's lab aims to elucidate the molecular mechanisms of non-coding RNAs and explore their diagnostic and therapeutic potential in treating human disease. We look forward to them joining us at our biannual Editor meeting in Oxford this year.

BiO has long been included in The Directory of Open Access Journals - a service that indexes high-quality, peer-reviewed Open Access research journals. BiO is now the proud owner of a DOAJ seal. This is a “mark of certification for Open Access journals, awarded by DOAJ to journals that achieve a high level of openness, adhere to best practice and high publishing standards”. In an age of proliferating ‘predatory’ Open Access journals, it is essential that authors have the means to identify journals in which they can publish with confidence.

BiO has complied with the following criteria to receive the seal:

- uses DOIs as permanent identifiers;
- provides DOAJ with article metadata;
- deposits content with a long-term digital preservation or archiving program;
- embeds machine-readable CC licensing information in articles;
- allows generous reuse and mixing of content, in accordance with a CC BY, CC BY-SA or CC BY-NC license;
- has a deposit policy registered with a deposit policy registry;
- allows the author to hold the copyright without restrictions.

First Persons have come to the fore of BiO. Our series of interviews with the first authors of a selection of papers published in BiO are now being published as articles in their own right (with their own DOI). These interviews are proving hugely popular with early-career researchers. This is just one of the ways in which BiO and The Company of Biologists give back to the biological sciences community, including scientific meeting grants, and funded places at Company workshops and meetings. BiO will also be appointing early-stage PIs to its Editorial Board team.

Mentoring early-career researchers in the art of peer review is crucial to the future of scientific publishing. BiO encourages the involvement of postdocs and other early-career scientists in the peer review process. We ask that the name of the co-reviewer is reported to the Editor (a field will be provided in the report form for this purpose).

BiO continues to receive increased numbers of submissions from all around the world. These articles must be rigorously reviewed by the community. We now publicly thank all our reviewers for their expertise and time, as well as our authors, readers and editors for their support (see supplementary information for a list of reviewers who completed a review for BiO in 2018).

## Supplementary information

Supplementary information available online at http://bio.biologists.org/lookup/suppl/doi:10.1242/bio.042119
